# Long-Term Hydration Study of Blended Cement: Calcined Kaolinite–Illite Composite Clays Compared to Fly Ash

**DOI:** 10.3390/ma18225123

**Published:** 2025-11-11

**Authors:** Matea Flegar Pregernik, Marijana Serdar

**Affiliations:** Department of Materials, Faculty of Civil Engineering, University of Zagreb, 10000 Zagreb, Croatia; matea.flegar@grad.unizg.hr

**Keywords:** limestone–calcined clay cement (LC^3^), low-kaolinitic clays, hydration, fly ash, sustainable binders

## Abstract

**Highlights:**

**What are the main findings?**

- Long-term hydration of low-grade calcined clays studied up to 1 year.
- Both kaolinite–illite clays showed similar hydration despite mineralogical differences.
- Visible synergy observed between calcined clays and limestone filler.

**What is the implication of the main finding?**

- Low-grade clays are viable raw materials for LC^3^ production.
- Comparable performance to fly ash system supports large-scale application.

**Abstract:**

Calcined clays are a promising route to lower-carbon binders, but widescale adoption of limestone calcined clay cements (LC^3^) requires using low-kaolinitic resources due to the limited availability and pricing of high-grade sources. This study evaluates the long-term hydration of two locally available kaolinite–illite composite clays (kaolinite contents 18% and 13%) in binary (30% SCM) and ternary LC^3^-type (30% SCM + 15% limestone) binders, benchmarked against OPC and fly ash systems. Over 1 year, thermogravimetric analysis showed lower portlandite (CH) and increasing bound water in SCM systems relative to OPC, reflecting ongoing secondary hydration reactions of the SCMs. XRD/Rietveld confirmed formation of hemi- and monocarboaluminate, enhanced in LC^3^ versus the corresponding binaries. The degree of hydration (DoH) for clay blends exceeded OPC from 7 days onward and reached comparable hydration levels after 1 year, indicating a beneficial later-age contribution from illite. Mortar tests showed that binary clay mixes approximated the 42.5 N class target at 28 days, while all LC^3^ mixes exhibited lower early strength but additional strength gain from 28 to 90 days. Overall, low-grade kaolinite–illite clays combined with limestone deliver LC^3^ systems with hydration development and mechanical performance comparable to commonly used fly ash-based cements, supporting their feasibility as regionally sourced, low-carbon binders.

## 1. Introduction

According to the United Nations Environmental Program Sustainable Building and Climate Initiative (UNEP-SBCI) [1], reducing the environmental impact of cement production relies primarily on increasing energy efficiency and lowering the clinker factor. While significant progress has been made in energy efficiency [2], reducing clinker content remains the key strategy. The use of supplementary cementitious materials (SCMs) as partial clinker replacements is well established, but high substitution levels (>25%) are still uncommon in practice [3]. To accelerate the transition toward sustainable binders, the European Committee for Standardization introduced EN 197-5, which enables commercialization of low-clinker cements (up to 60% replacement) under the designations CEM II/C-M and CEM VI [4,5].

Industrial by-products such as fly ash and blast furnace slag have been widely used as SCMs [6,7,8], but their availability is declining. Coal-based power generation in the EU has dropped by one third since 2012 [9], and steel production has decreased in recent years while raw material prices have increased [10]. This shortage highlights the need for alternative, abundant sources of SCMs.

Calcined clays represent a promising option. Thermal activation disrupts their crystalline structure, generating amorphous phases with enhanced pozzolanic reactivity. The reactivity depends on mineralogy, bond strain, heating regime, and impurities [11]. In limestone–calcined clay cements (LC^3^), limestone not only provides filler effects but also reacts synergistically with alumina from SCMs to form carboaluminate hydrates. These contribute to pore refinement, densification, and increased tortuosity, leading to improved durability and resistance to chloride ingress [12,13,14].

Although extensive research has examined calcined clays and LC^3^ systems, limited work has focused on low-grade composite clays containing both kaolinite and illite, which are naturally available and can also be sourced from quarry waste. Msinjili et al. investigated illite-bearing clays from brick production and compared their mineralogy, calcination behaviour, and durability performance in cement blends with that of kaolinitic clays [15,16]. They observed that the shift of the 004 reflection of illite during calcination indicated the extent of dehydroxylation, which occurred gradually between 650 and 850 °C, and that incomplete dehydroxylation at 650 °C limited the transformation of illite. At higher temperatures (≥850 °C), sintering led to increased particle size and reduced specific surface area. Notably, an illitic (brick) clay with a lower Fe_2_O_3_ content, calcined at 850 °C, performed comparably to kaolinitic clays in terms of strength and reactivity at a 15 *v*/*v*% replacement level. Moreover, calcined illitic and mixed kaolinitic–illitic clays demonstrated improved chloride penetration resistance due to pore structure refinement from pozzolanic reactions, though carbonation resistance was somewhat reduced, consistent with other SCM-based systems. Recent findings also indicate that while illite exhibits lower initial reactivity, it contributes to strength gain at later stages of hydration, confirming its potential as a viable component in LC^3^ systems [17]. Furthermore, studies on composite cements containing calcined illite clay and limestone filler have shown that their combination promotes the formation of calcium hemi- and monocarboaluminate phases at later ages, enhancing microstructural densification and contributing to long-term strength development [18]. Recent work by Atasever and Erdoğan [19] systematically examined the influence of both clay type (kaolinite, illite, montmorillonite) and component fineness on the hydration and strength development of LC^3^ cements. Their results showed that finer calcined clays, especially kaolinitic ones, promoted the formation of hemicarboaluminate and monocarboaluminate phases and enhanced later-age strength. Illite-based LC^3^ systems exhibited larger pore sizes and lower normalized cumulative heat, confirming their lower reactivity but continued contribution to hydration at later ages.

The research thus showed promising potential for the use of low-grade clays as supplementary cementitious materials at a low replacement level but lacked an in-depth investigation of hydration mechanisms at higher replacement ratios, particularly those with lower kaolinite and higher illite content, and in combination with limestone. Investigating such materials is crucial for expanding LC^3^ applications and ensuring large-scale feasibility of sustainable cements.

In this study, two locally available clays from Croatia with relatively low kaolinite content (up to 18%) were investigated, alongside fly ash and limestone filler, to evaluate their potential in LC^3^-type cements. The experimental programme was designed to evaluate the hydration behaviour, microstructural development, and mechanical performance of the selected binders over short and long curing periods. Particular attention was given to the role of sulphate balance, portlandite evolution, and the formation of hydration products in binary and ternary systems, with emphasis on the role of illite in the hydration process.

## 2. Materials and Methods

### 2.1. Raw Materials Preparation

**Figure 1:**
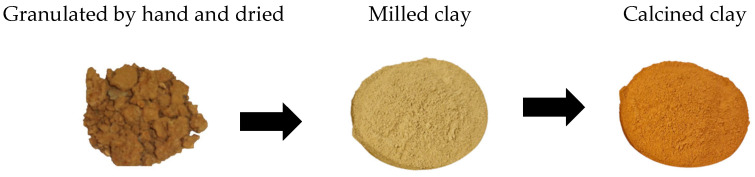
Photographs of Clay_a in various stages of material preparation.

The raw SCMs (fly ash and clays) were first dried in a laboratory oven at 60 °C for 24 h. The clays were then ground in a disc mill for 90 s and calcined at 800 °C for characterization and before mixing the pastes. The binary and ternary binders were pre-mixed and dried by hand. The mixing proportions are presented in Table 1. The chemical and mineralogical data of the selected raw materials, as well as the XRD patterns of the raw clay, are provided in the Supplementary Data (Table S1 and Figure S1). Two types of locally available natural clays were used in this study, both with relatively low kaolinite content. The clay with the highest kaolinite content in the region comes from a brick factory in Maruševec, Croatia (Clay_a), while the other is a clay from near one of the most important cement factories in Croatia, in Našice (Clay_b). Both clay materials have similar chemical but slightly different mineralogical compositions, with a kaolin content of 18 and 13% for Clay_a and Clay_b, respectively. Both clay samples have impurities, with Clay_b having a relatively high proportion of quartz but a lower proportion of muscovite compared to Clay_a. Illite was found in both samples, although Clay_b had a significantly higher proportion. The relatively high proportion of iron oxides in both clays influences the reddish colour of the calcined product, Figure 1. The fly ash sample corresponds to the characterized sample FA_T, originating from Tuzla, Bosnia and Hercegovina. It was classified as class F even though it has a higher CaO content than usual, higher than 10%. The limestone powder for the study was used as limestone quarry waste from a local aggregate producer, with a CaCO_3_ content of 88% (determined by XRD; detailed mineralogical composition provided in the Supplementary Data). For this reason, the purity in terms of calcium carbonate is slightly lower and the D50 value of the particle size distribution slightly higher than for the commonly used limestone filers [20,21,22].

A detailed explanation of the raw material characterization techniques and results can be found in a previous publication by the authors [23].

### 2.2. Cement Paste Preparation

Cement paste samples were prepared using deionized water, 500 g of dry binder and a 0.5 w/b ratio in a high-shear laboratory mixer at 1600 rpm for 2 min. The mixture was poured into cylindrical plastic containers and kept sealed at 20 °C for 24 h. The cement paste samples were then transferred to a slightly wider container with the minimum amount of water sufficient to submerge the cement paste. The samples were then cured at 20 °C until testing. When the test age was reached, the samples were removed from the curing containers and 3 to 5 mm thick slices were cut out of the paste cylinder for testing. The top and bottom of the samples were discarded. For the TGA measurements, hydration was stopped using the solvent exchange method described in [24] by immersing the paste discs in isopropanol. The 1-day-old samples were immersed for 24 h, while the older samples were kept for up to 7 days (as recommended in [24]). After solvent exchange, the discs were placed in a vacuum desiccator for at least 48 h before testing.

### 2.3. Testing Methods

Isothermal calorimetry was used as a tool for sulphate adjustment of LC3 and other mixed systems. Prior to testing, all materials were stored in airtight containers and conditioned at 20 ± 2 °C. The dry materials (SCM, cement, and gypsum) were first weighed and mixed, after which water was added to achieve a water-to-cement ratio of 0.5. The pastes were mixed in a high-shear mixer (as in the other tests) to obtain a homogeneous mixture. The fresh pastes were then immediately cast into sample containers and placed in an isothermal calorimeter (TAM Air, TA Instruments, New Castle, DE, USA) maintained at 20 °C. The heat evolution was recorded continuously for 7 days (168 h).

With a high specific surface and additional alumina, metakaolin evokes reactivity of the system. If there is not enough sulphate in the system, the aluminate reaction (visible as a peak in the calorimetry curve) occurs earlier, with a higher intensity and a narrower peak than in OPC system [14]. If enough gypsum is present, the C_3_S reaction occurs earlier or the aluminate peak is delayed, which separates the two peaks from each other. More gypsum is added, a prolongation of the C_3_A reaction is observed, shifting the second peak to the right and becoming lower and broader [22].

Thermogravimetric analysis was used to study hydration evolution—through portlandite consumption and bound water content. After hydration stoppage, a piece of the cut-out disc sample was crushed in a laboratory agate mortar. Immediately afterwards, 50 mg of the finely pulverized sample was placed in a special aluminum crucible to minimize the influence of carbonation. The sample was heated from 30 to 1000 °C at a rate of 10 °C/min under an N2 flow of 30 mL/min. The measurement was performed with a Mettler–Toledo TGA/SDTA 851 instrument. The content of bound water (H) and portlandite (CH) was calculated using the tangent method by measuring the mass loss in the temperature intervals between 40 and 550 °C for bound water and 450 to 550 °C for portlandite. The lower limit of 40 °C was selected to exclude the evaporation of physically adsorbed (free) water, which occurs below this temperature, while the mass loss above 40 °C corresponds primarily to dehydration of hydrates such as C–S–H and AFt/AFm phases. The upper limit (550 °C) was chosen to avoid interference from CaCO_3_ decarbonation, which begins above approximately 600 °C. This approach follows established methodologies reported in [24]. The results are normalized to 100 g anhydrous and calculated according to the following Equations (1) and (2).

(1) $$H=\frac{W_{40}-W_{550}}{W_{550}},$$

(2) $$CH=\frac{W_{450}-W_{550}}{W_{550}}\times \frac{74}{18},$$

X-ray diffraction was used also to identify and quantify the phase composition of hydrated pastes. The measurement was performed on powders of dry mixes and freshly cut disc samples to monitor the evolution of hydration products. The scan ranged from 7 to 70° with a step size of 0.0167° 2θ and a time per step of 30 s, with scan times of approximately 15 min. Quantitative analysis of the phases for dry powders and pastes was carried out using Rietveld refinement [25], using X’Pert HighScorePlus Academic software, with rutile powder (Kronos-2300 titanium dioxide) as an external standard to quantify the amorphous phase. The external method identifies the weight fractions of the phases by comparing the refined phase scale factor with the external rutile standard measured under the same conditions [26]. The accuracy of the quantification of phases in anhydrous mixtures and hydrated cement pastes depends on the parameter variations chosen, such as the preferred orientation, unit cell parameter, etc. [24] to ensure the best possible fit. While these factors have been carefully managed, the focus of this work is to compare a large number of samples that have been controlled and treated and analyzed in the same way.

Based on the phase quantification obtained, a degree of hydration (DoH) quantification was performed for each sample [27]. In order to compare the results of anhydrous powders with freshly cut disc samples, the results of the Rietveld analysis of discs were rescaled by correcting the calculated values with the water–binder ratio of the paste [24]. The recalculated, normalized results were determined using the following formulas:

(3) $$W_{j,rescaled}=\;W_{j,Rietveld}\;(1+\frac{w}{b}),$$

(4) $${DoH\;(\%)}_{t}=\left(\frac{\sum {\left(C_{3}S,C_{2}S,C_{3}A,C_{4}AF\right)}_{t0}-\sum {\left(C_{3}S,C_{2}S,C_{3}A,C_{4}AF\right)}_{t}}{\sum {\left(C_{3}S,C_{2}S,C_{3}A,C_{4}AF\right)}_{t0}}\right)\times 100$$

The DoH is calculated from the mass fractions of the reacted C_3_S (Alite), C_2_S (Belite), C_3_A (Aluminate), and C_4_AF (Ferrite) (Equation (4)), where *t*_0_ stands for the initial state (anhydrous) of clinker phases and *t* is the time of interest of the remaining clinker phases.

Backscattered electron imaging (SEM-BSE) and energy dispersive X-ray spectroscopy (SEM–EDS) were performed using an FEI Quanta 200 (Thermo Fisher Scientific, Hillsboro, OR, USA) equipped with a Burker XFlash 4030 detector to monitor hydration products. Cement paste samples were prepared following standard procedures for impregnation, polishing, and coating. Small disc fragments were hand-polished, vacuum-impregnated with transparent epoxy, and mounted in rubber moulds. Pre-polishing was done with P1200 grit sandpaper, followed by automated polishing (20 N, 150 rpm) using petroleum lubrication and diamond sprays of 9, 3, and 1 µm, each applied for 45 min. Samples were ultrasonically cleaned between steps, carbon-coated, and stored in a desiccator until analysis.

Compressive strength test of mortars was also tested according to the procedure described in the standard EN 196-1 [28]. A constant 0.5 water-to-binder ratio was used for mortar preparation with standardized sand as aggregate. Each mixture was mixed in one batch from which 40 × 40 × 160 mm samples were cast. After casting, samples were held covered in laboratory conditions for 24 h, followed by demoulding and curing in a humidity chamber until testing time. Compressive strength tests were performed on two prisms for each mix at the specified curing ages, following EN 196-1 [28].

An average of two samples was tested for each method, including calorimetry, thermogravimetric analysis (TGA), and compressive strength testing. One paste sample was prepared for XRD and SEM analyses. Microstructural observation was performed using SEM in backscattered electron (BSE) mode, while elemental composition was analyzed using energy-dispersive X-ray spectroscopy (EDS). For SEM–EDS analysis, three micrographs were recorded at each selected magnification, and approximately 30 points were analyzed for EDS measurements. Detailed images are presented in Figure S4 of the Supplementary Data.

## 3. Results and Discussion

### 3.1. Sulphate Balance

Blended cements often require an optimal amount of sulphate addition, which differs from OPC. If the sulphates are not well adjusted, this can cause the aluminate peak to collide with the alite peak, which then lowers the heat-flow curve [22] and thus affects the hydration reaction. It has been shown that the addition of gypsum influences the separation of the two peaks, which can lead to an increase in compressive strength in the first 24 h of clay cements containing limestone cements [14,29]. The same was also found for fly ash and limestone [30].

**Figure 2:**
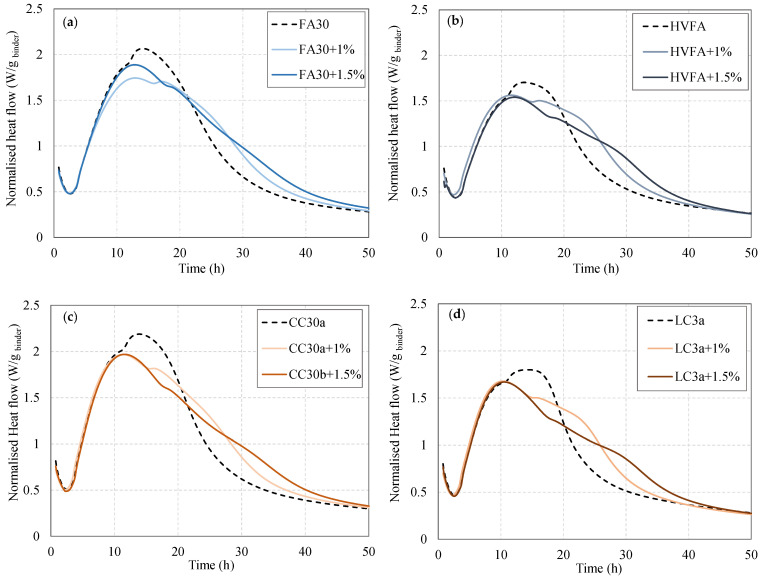
Sulphate adjustment for cement blends containing (**a**) fly ash, (**b**) fly ash and limestone, (**c**) calcined clay, and (**d**) calcined clay and limestone binders.

Figure 2 shows the normalized heat flow per binder content for the four selected blended systems (FA30, HVFA, CC30a, and LC3a). The results show differences in hydration performance when 0, 1, and 1.5% gypsum is added to the mixes. It can be observed that the mixtures without limestone (FA30 and CC30a) show an almost complete merging of the two peaks when no gypsum is added. The addition of 1% gypsum separates the peaks and reduces the heat flow of the aluminate peak. Similar behaviour is observed for the limestone mixtures, but the first alite peak appears to increase slightly when sulphate is added. For both the HVFA and LC3 systems, the difference between the alite and aluminate peaks is more pronounced when 1% gypsum is added. The addition of 1.5% gypsum reduced the intensity of the first heat flow peak and broadened the aluminate peak in all systems, indicating over-sulphation in this case. For this reason, the addition of 1% gypsum was considered for the next test phase for all mixtures.

### 3.2. Hydration Development and Portlandite Evolution

**Figure 3:**
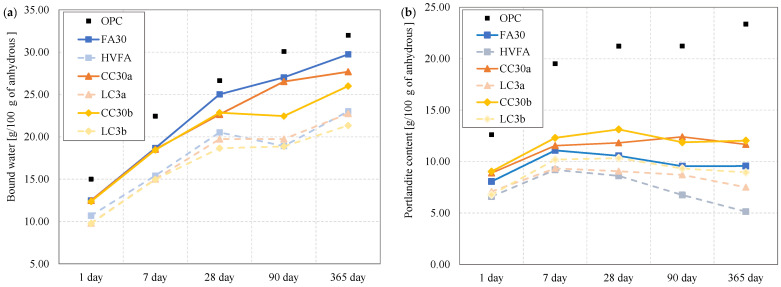
Normalized (**a**) bound water and (**b**) portlandite content obtained by TGA measurement. Note: The connecting lines between data points serve solely to indicate overall trends and do not represent intermediate data.

The hydration behaviour of the various binders is different, especially when comparing binders containing OPC and SCM. The hydration products in the cement paste also differ between binary and ternary binders. Figure 3 presents the bound water and portlandite content attained from TGA measurements.

The bound water measurements in the diagram Figure 3a show a similar trend of hydration development for all samples, increasing with time. From the first day onward, the bound water content as well as the portlandite content was lowest for ternary blends, intermediate for pastes with binary blends, and highest for the plain Portland cement paste. The binary blends (FA30 mostly, followed by CC30a and then CC30b) mimic the OPC behaviour in bound water content after 28 days and up to 1 year, indicating a similar reactivity. The ternary blends show lower bound water amounts from the start.

The portlandite content in Figure 3b differs considerably. It is known that the main product of hydration of ordinary Portland cement is portlandite, and the amount of portlandite in OPC increases with time. The pozzolanic effect that occurs in SCM binders manifests itself in the consumption of portlandite to form other hydrates. From the diagram in Figure 3b it can be seen that fly ash binders consume more portlandite in both binary and ternary systems. Both calcined clay samples appear to hydrate in a similar way, in both tested systems—alone and with limestone. The decrease in portlandite is more pronounced after 28 days, indicating a slower pozzolanic response of the SCM systems than the OPC mixture.

The bound water content determined from TGA represents the mass loss associated with the dehydration of hydrates such as C-(A-)S-H, AFt, and AFm phases within the temperature range of 40–550 °C. Consequently, higher bound water values correspond to a larger quantity of hydration products formed, indicating a greater degree of hydration and pozzolanic reactivity of the binder. The enhanced long-term bound water and reduced CH contents observed in this study are consistent with the findings of Zhao et al. [14], who reported that thermally activated illitic materials exhibit improved pozzolanic activity due to structural dehydroxylation and partial amorphization. Their results support the interpretation that illite, though less reactive initially, contributes to ongoing hydration and strength development at later ages. Similar interpretations have been reported in previous studies, where the bound water and portlandite contents decreased with increasing cement replacement due to dilution, yet their relative levels reflected the ability of the supplementary cementitious material to generate hydrates through secondary reactions [15].

Some suggest [31,32] the portlandite content calculated from TGA should be corrected by the mass loss corresponding to the CaCO_3_ decomposition caused by the carbonation of samples. This procedure was not applied in this case due to the significant amount of limestone filler in anhydrous cement and SCMs that cannot be clearly distinguished from the amount of carbonated portlandite. All samples were stored and tested in the same manner, to minimize the carbonation effect.

### 3.3. Other Hydration Products

The change in the proportions of the different hydration products formed with the addition of SCM (calcium aluminate silicate hydrates, ettringite, and carboaluminates) can influence the mechanical and other properties of hardened paste. For that reason, hydration products were monitored by quantitative XRD measurements. Due to the extensive amount of data, diagrams of the calculated phases for all samples are shown in the Supplementary Data (Figure S2).

**Figure 4:**
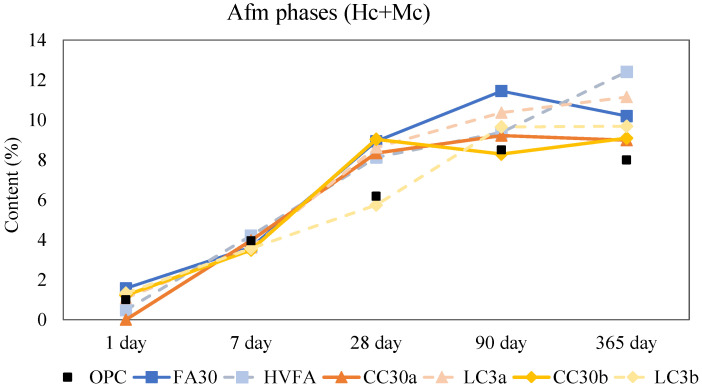
Normalized content of hemi- and monocarboaluminates. Note: The connecting lines between data points serve solely to indicate overall trends and do not represent intermediate data.

After a day of hydration, the main hydration products for all mixes were portlandite, ettringite, and amorphous phase. For both binary and ternary blends, the amounts of hemicarbo- and monocarboaluminates were slightly evident at this early stage. Figure 4 shows the calculated and normalized amounts of hemi- and monocarboaluminates. After one year of age, the addition of limestone promoted the formation of hemi- and monocarboaluminates, which increase in the total amount for blends containing additional limestone (HVFA, LC3a and LC3b) compared to their binary variants (FA30, CC30a, and CC30b). There was a slight decrease visible from 90 days to 1 year of age for the OPC and fly ash binary samples, as shown in the XRD quantification (Figure S3 in the Supplementary Data). Some studies suggest that this decline may be due to the transformation of monocarboaluminate into other aluminosilicate hydrates or hydrogarnet phase [33,34]. However, since these phases were not detected in the present XRD analysis, the most likely explanation is that monocarboaluminate decomposes, either due to carbonation or ongoing hydration. This interpretation is supported by a corresponding increase in the amorphous content of these samples over the same period. It is possible that other amorphous products form during this decomposition process, such as amorphous aluminum hydroxide (AH_3_) [35].

Interestingly, there is no sight of monosulfoaluminates, neither in the SCM blends nor OPC. This is probably due to the high calcite content in the anhydrous OPC which stabilizes ettringite and thus favours carboaluminate instead of sulfoaluminate formation. The free sulphates become more available to form ettringite in later stages [14]. Strätlingite was not observed in any of the calcined clay samples which is in agreement with other research and models for low kaolinite content clays (<40%) [36,37]. It has been implied that the lack of alumina in illitic clays could prevent strätlingite formation [38].

**Figure 5:**
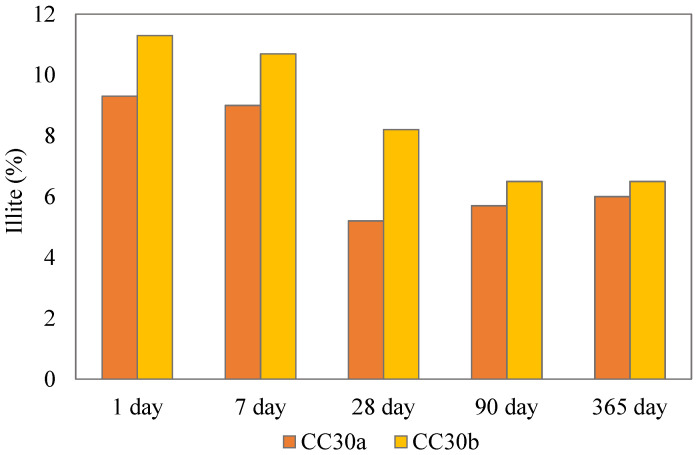
Illite content in clay containing paste samples.

The intensities of illite peaks for cement paste systems declined over time suggesting the pozzolanic activity of the partially calcined illitic clay. Figure 5 shows the calculated illite content in the paste samples that contained calcined clay over the period of 1 year of hydration. The decline was most pronounced in the higher illite bearing clay CC30_b s after 7 days of hydration, suggesting that calcined illite actively influences the hydration process in Portland cement composites. However, it is important to note that Rietveld analysis of minor phases can produce variable results. This is evident in the CC30a sample, where the calculated illite content at 28 and 180 days appeared slightly lower than at later stages. Still, the overall downward trend is consistent across all systems.

### 3.4. Degree of Hydration

**Figure 6:**
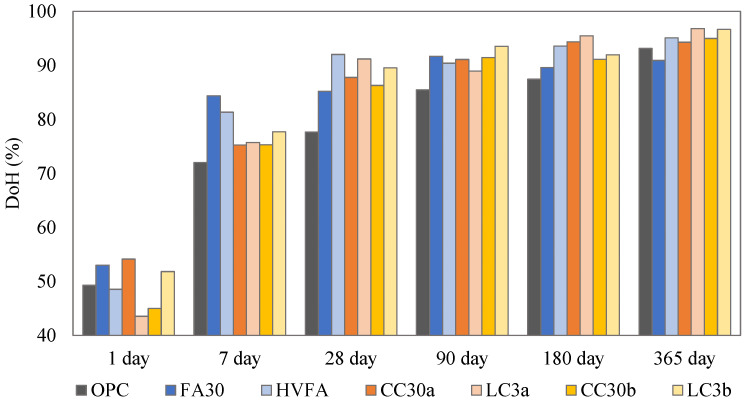
DoH for all mixes.

In order to determine the long-term reactivity of the selected binders, the DoH was calculated taking into account the reacted cement (in Supplementary Data) (Figure 6). From an early age (1 day), the FA30 and the CC30a samples showed a higher DoH than the OPC reference, while after 7 days all SCMs exceeded the OPC reference. This was due to the filling effect of the added SCMs [27], which is particularly visible in the samples containing limestone; they showed a higher DoH than the binary mixtures. The samples with fly ash, both in the binary and ternary systems, showed rapid reactivity for up to 28 days (when they exhibited the highest DoH), after which their hydration became almost constant. For all systems containing Clay_a and Clay_b the DoH increased gradually with time. The slightly higher kaolinite content of Clay_a seemed to have an effect for up to 90 days, when the DoH was higher. For up to 365 days, both clay systems showed a similar DoH. This result supports the earlier conclusions that illite adds to the reactivity at later stages.

### 3.5. SEM

**Figure 7:**
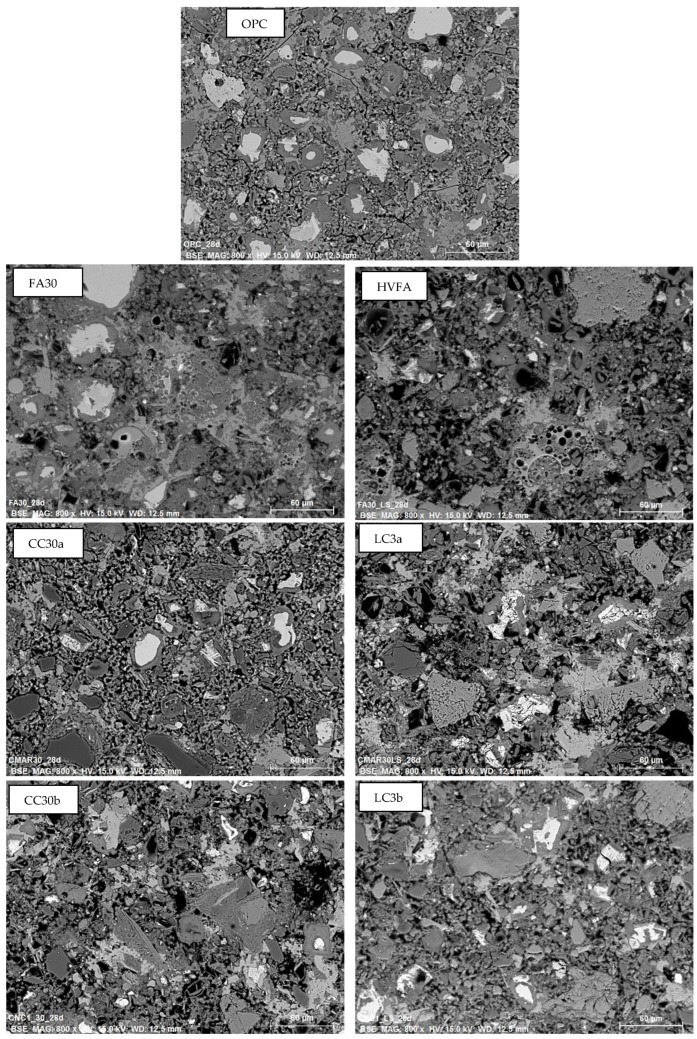
SEM BSE low magnification micrographs of 28-day-old cement pastes.

Figure 7 shows the same microscopic images with lower magnification of 28-day-aged samples to compare the structure of selected paste mixtures. In the OPC sample, a clear distinction can be seen between the unhydrated cement grains, the inner product, and the portlandite. Samples containing 30% SCM show a slightly denser matrix after 7 days than the OPC sample, which is due to the additional AFm and other phases that fill the gaps and form the outer product (OP). The CC30a sample appears to be the most heterogeneous and compact, which is due to the addition of alumina and silica. This is consistent with recent studies on binary and ternary mixes containing clay and metakaolin [39]. The same cannot be said of the HVFA and LC3 samples. Larger limestone (and quartz) particles, which affect the hydration time, are still visible.

### 3.6. Compressive Strength

**Figure 8:**
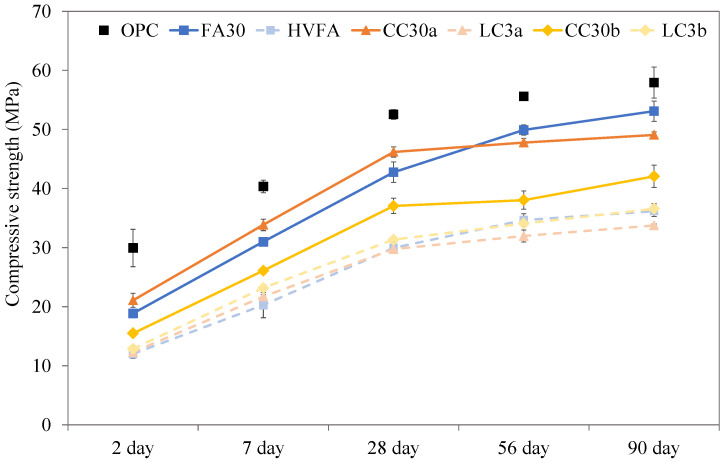
Compressive strength of mortars after 2, 7, 28, 56 and 90 days. Note: The connecting lines between data points serve solely to indicate overall trends and do not represent intermediate data.

The compressive strength of mortar samples up to 90 days of age is presented in Figure 8, the error bars in represent the standard deviation calculated from the four halves of the prisms tested in compression.

Although the Portland cement binder used is CEM I 42.5 N, the OPC results show much higher 28 day strength than required. Samples FA30 and CC30a exhibit a strength closer to the standardized requirements of 42.5 N after 28 days for these types of cements. In contrast to most fly ash systems that show early strength development, the present FA30 system demonstrates the same strength as CC30a after 7 days. This was clearly due to the higher lime content of the fly ash used which interferes with the reactivity [40].

All samples that contained additional limestone showed lower strengths, though the strength increase in later stages (from 28 to 90 days) seems to be higher than for their binary versions. LC3b appears to slightly exceed the strength of LC3a in later stages, which is comparable to the results of DoH.

## 4. Conclusions

This study examined the hydration behaviour and microstructural development of binary and ternary cementitious binders containing low-grade calcined clays and limestone waste, comparing their performance to fly ash systems and ordinary Portland cement (OPC). The analysis spanned up to one year and focused on the degree of hydration, reaction products, and mechanical performance.

The hydration products formed through pozzolanic and filler reactions included carboaluminates, with limestone enhancing the development of hemicarboaluminates and monocarboaluminates over monosulfoaluminates. These trends align with the behaviour of systems containing low-grade calcined clays. Through the studied microstructural characterization, it was proven that even low-kaolinite and illite bearing calcined clays (Clay_a and Clay_b) exhibited similar effects. It can be assumed that the illite content in these clays contributed to long-term reactivity, as evidenced by its gradual consumption over one year.

In terms of hydration behaviour, binary and ternary mixes containing SCMs exhibited lower portlandite content compared to OPC, reflecting significant pozzolanic activity. Ternary blends containing limestone showed enhanced hydration due to synergistic filler effects. Measurements of the degree of hydration (DoH) revealed steady increases for clay-containing systems, with illite contributing to hydration at later stages.

Microstructural observations using scanning electron microscopy (SEM) indicate a denser paste matrix in systems containing calcined clays, which was attributed to additional silica and alumina from the clays. However, ternary blends with added limestone quarry waste displayed significant amounts of unreacted particles after 28 days. This could be due to the high carbonate amount intermixed in the clay and fly ash particles, and even in the OPC mix.

The mechanical performance of binary SCM-containing samples was comparable to OPC, with compressive strength development observed in all cases. Ternary blends containing limestone exhibited slower early strength development but demonstrated increase in strength at later stages. The addition of limestone reduced early strength but added to long-term mechanical performance. Among the samples, LC3b (containing Clay_b) performed slightly better than LC3a (containing Clay_a).

This study suggests that both investigated low-grade calcined clays show promising potential as raw materials for the production of LC^3^-type cements. Despite differences in mineralogical composition, their performance in terms of hydration, microstructure, and mechanical properties was comparable, indicating their suitability as potential alternatives to conventional SCM-containing binders. These results are particularly relevant in regions where high-grade kaolinitic clays are scarce or economically impractical, demonstrating that locally available low-grade clays can provide a practical and sustainable option for cement production. By leveraging the synergy between calcined clay and limestone, effective optimization of hydration and binder performance can be achieved even with clays of relatively low kaolinite content.

It should be emphasized that this study did not address long-term durability aspects, which remain essential for a complete evaluation of these binders. Future work should therefore focus on durability-related properties and environmental assessment to further confirm the large-scale applicability of low-grade clays in LC^3^ systems.

## Figures and Tables

**Table 1:** Nomenclature and mixing proportions of tested paste samples in mass (g).

Nomenclature	Replacement Ratio	FA	CC	Limestone Powder	Cement
**OPC**	-	-	-	-	500
**FA30**	30%	150	-	-	350
**HVFA**	45%	150	-	75	275
**CC30a**	30%	-	150	-	350
**LC3a**	45%	-	150	75	275
**CC30b**	30%	-	150	-	350
**LC3b**	45%	-	150	75	275

## Data Availability

Data available in a publicly accessible repository: The data has been shared via FigShare DOI: 10.6084/m9.figshare.30257356, link: https://figshare.com/s/7a6a648d63531421bd1d, accessed on 30 September 2025.

## Supplementary Materials

The following supporting information can be downloaded at https://www.mdpi.com/article/10.3390/ma18225123/s1.
