# Supplementary material for: Long-Term Hydration Study of Blended Cement: Calcined Kaolinite–Illite Composite Clays Compared to Fly Ash
Source: Materials (Basel). 2025 Nov 11;18(22):5123. doi: 10.3390/ma18225123 (PMC12654212; doi:10.3390/ma18225123)
Supplement: Supplementary file 1 [file materials-18-05123-s001.zip › materials-3933413-supplementary.pdf]

Supplementarry data

*Table 1 Chemical and mineralogical composition of dry raw materials*

| Properties | | Cem I | FA | Clay_a | Clay_b | LS |
|---|---|---|---|---|---|---|
| Chemical composition (%) | CaO | 64.04 | 11.52 | 2.17 | 2.57 | 71.59 |
| | $SiO_2$ | 19.32 | 53.28 | 62.41 | 63.70 | 20.21 |
| | $Al_2O_3$ | 4.86 | 19.11 | 21.35 | 19.53 | 4.32 |
| | $Fe_2O_3$ | 2.94 | 9.05 | 7.26 | 6.80 | 1.43 |
| | MgO | 1.83 | 2.78 | 1.78 | 2.34 | 1.69 |
| | $Na_2O$ | 0.23 | 0.26 | 1.05 | 1.26 | 0.01 |
| | $K_2O$ | 0.82 | 1.51 | 2.50 | 2.52 | 0.15 |
| | Other | 5.92 | 2.49 | 1.47 | 1.31 | 0.61 |
| $D_{50}$[a] | | 9.95 | 15.24 | 10.7 | 13.4 | 18 |
| Kaolinite content of raw clay (%. TGA) | | | | 17.6 | 13.4 | |
| Illite content (%. XRD) | | | | 1.3 | 13.7 | |
| Mineral composition (%. XRD) | | | Quartz 5.4<br>Mullite 2.2<br>Hematite 0.3<br>Other 2.9 | Quartz 21.7<br>Muscovite 27.3<br>Vermiculite 2.4<br>Sanidine 13.6 | Quartz 44.2<br>Muscovite 10.7<br>Albite 11.2<br>Vermiculite 1.3<br>Clinochlore 0.2<br>Others 2.4 | Dolomite 1.5<br>Calcite 86.8<br>Vaterite 0.3<br>Serandite 3.2 |
| Amorphous content (%) | | | 89.3 | 14.3 | 11.6 | |

[a]Median particle size determined by laser diffraction using wet dispersion in 0.01 wt.% PAA (pH 10)

The diffractograms of calcined clays are shown in Fig. 1.

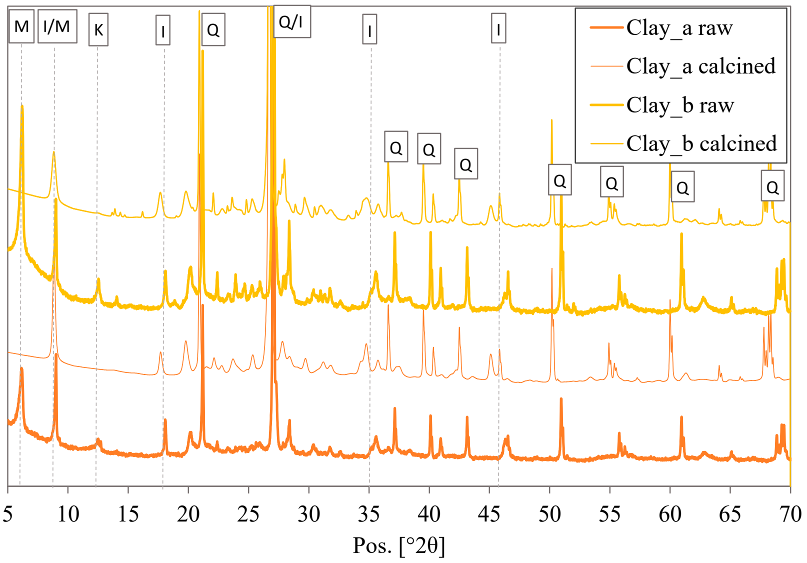


**Figure 1.** XRD patterns of raw and calcined clay cement where I:Illite, Q: Quartz, M: Muscovite, K:Kaolinite.

Normalised Rietveld analysis results of samples as anhydrous cement blends and pastes after 1, 7, 28 , 90 and 365 days is shown in Figure 2.

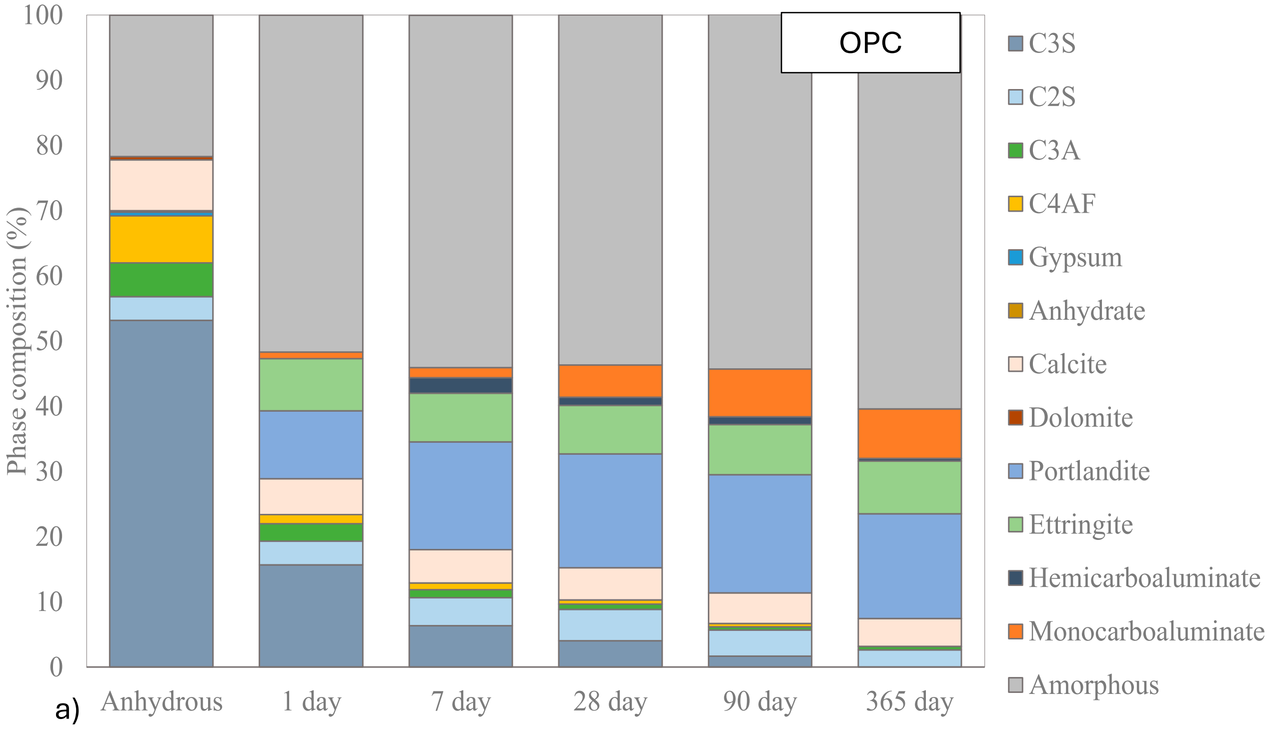


a)

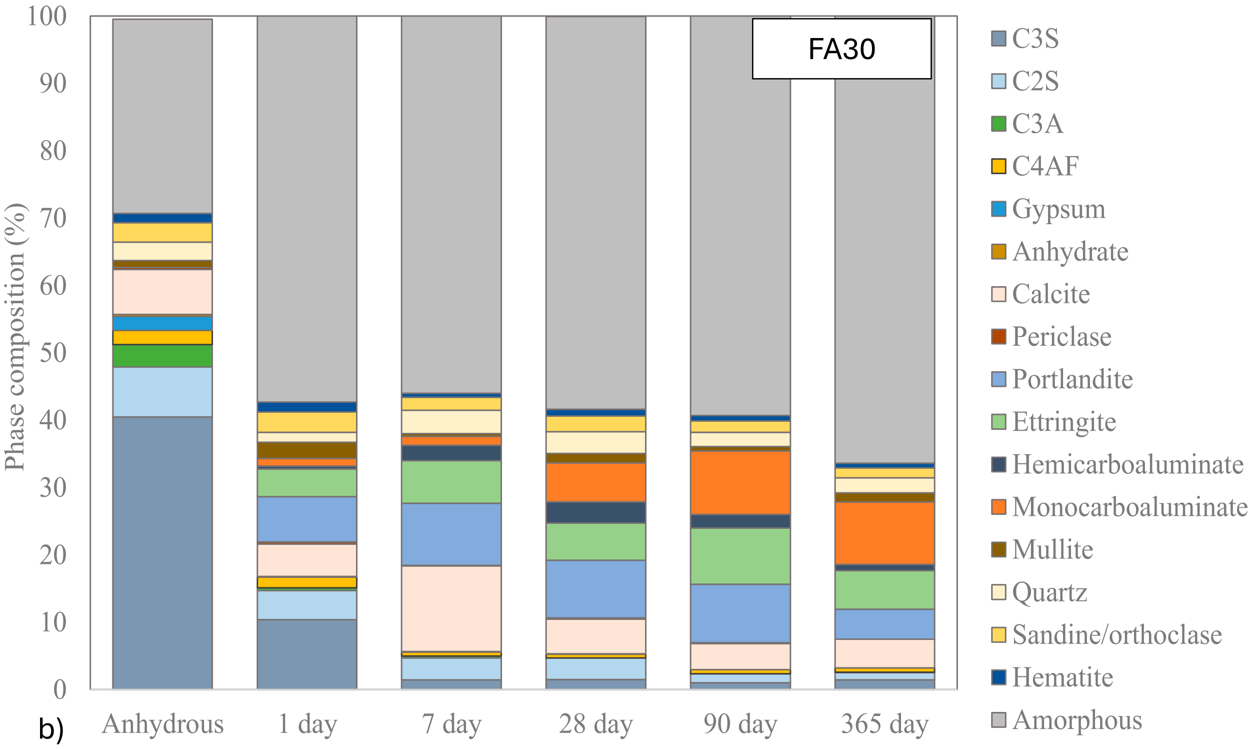


b)

c)

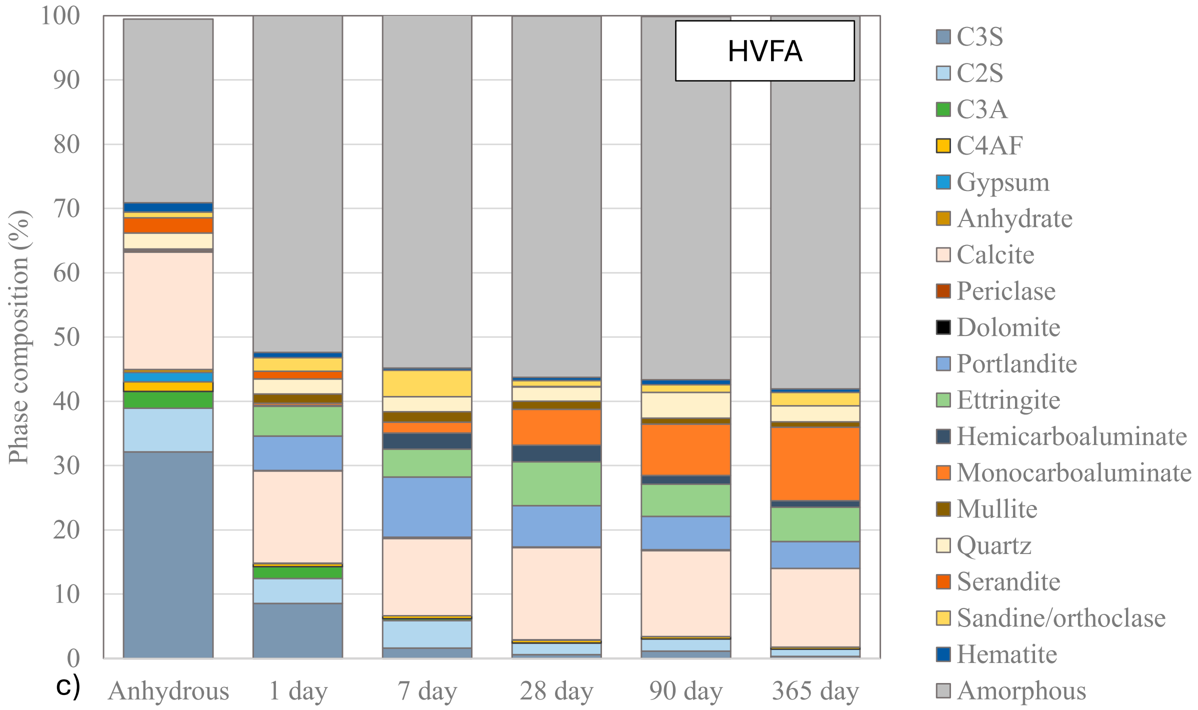
HVFA
Phase composition (%)
100
90
80
70
60
50
40
30
20
10
0
Anhydrous
1 day
7 day
28 day
90 day
365 day
C3S
C2S
C3A
C4AF
Gypsum
Anhydrate
Calcite
Periclase
Dolomite
Portlandite
Ettringite
Hemicarboaluminate
Monocarboaluminate
Mullite
Quartz
Serandite
Sandine/orthoclase
Hematite
Amorphous

d)

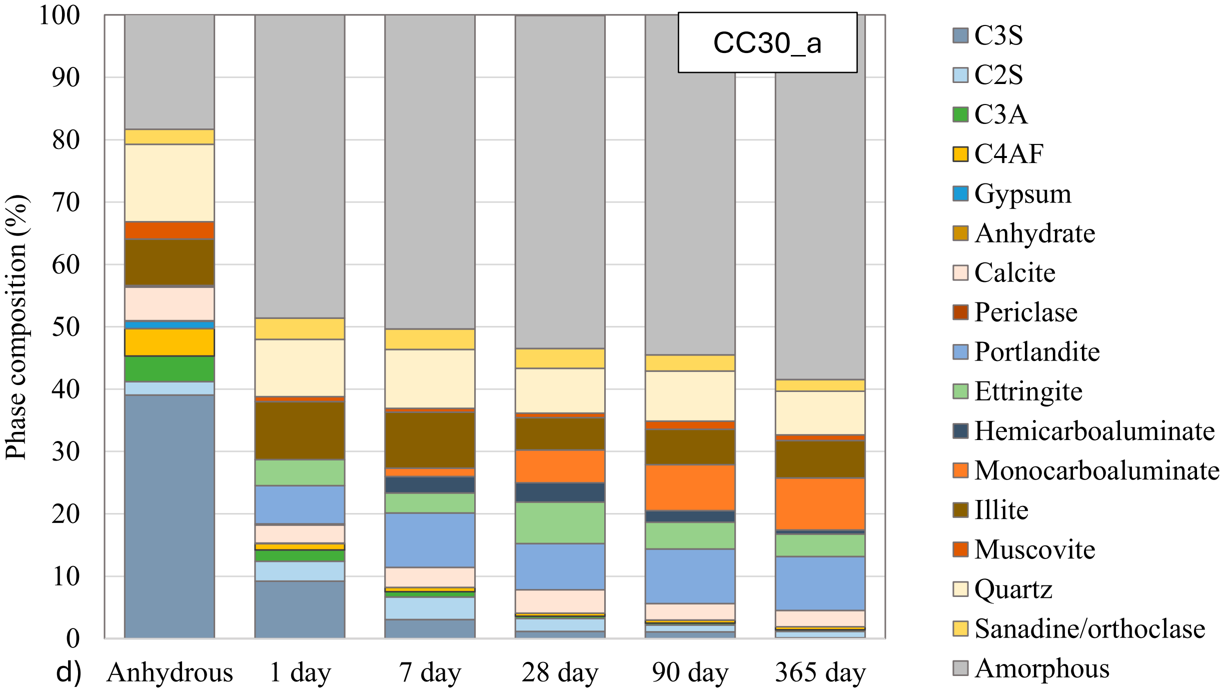
CC30_a
Phase composition (%)
100
90
80
70
60
50
40
30
20
10
0
Anhydrous
1 day
7 day
28 day
90 day
365 day
C3S
C2S
C3A
C4AF
Gypsum
Anhydrate
Calcite
Periclase
Portlandite
Ettringite
Hemicarboaluminate
Monocarboaluminate
Illite
Muscovite
Quartz
Sanadine/orthoclase
Amorphous

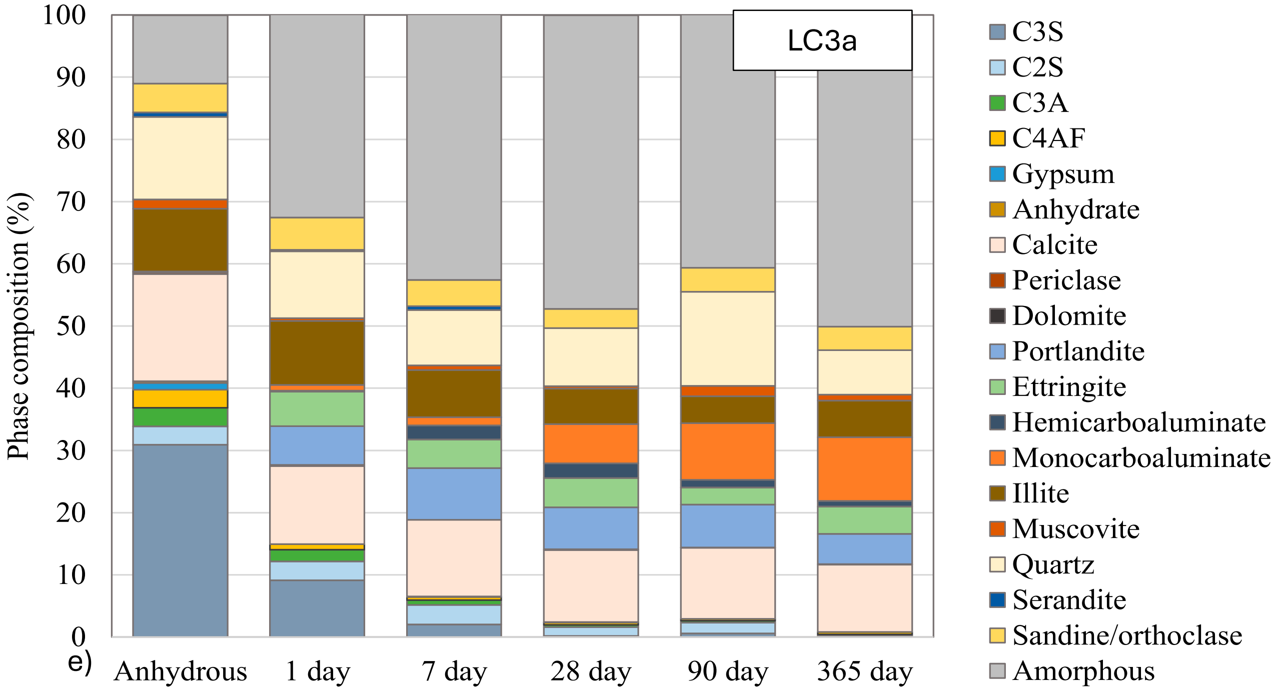

LC3a
Phase composition (%)
100
90
80
70
60
50
40
30
20
10
0
Anhydrous
1 day
7 day
28 day
90 day
365 day
C3S
C2S
C3A
C4AF
Gypsum
Anhydrate
Calcite
Periclase
Dolomite
Portlandite
Ettringite
Hemicarboaluminate
Monocarboaluminate
Illite
Muscovite
Quartz
Serandite
Sandine/orthoclase
Amorphous


e)

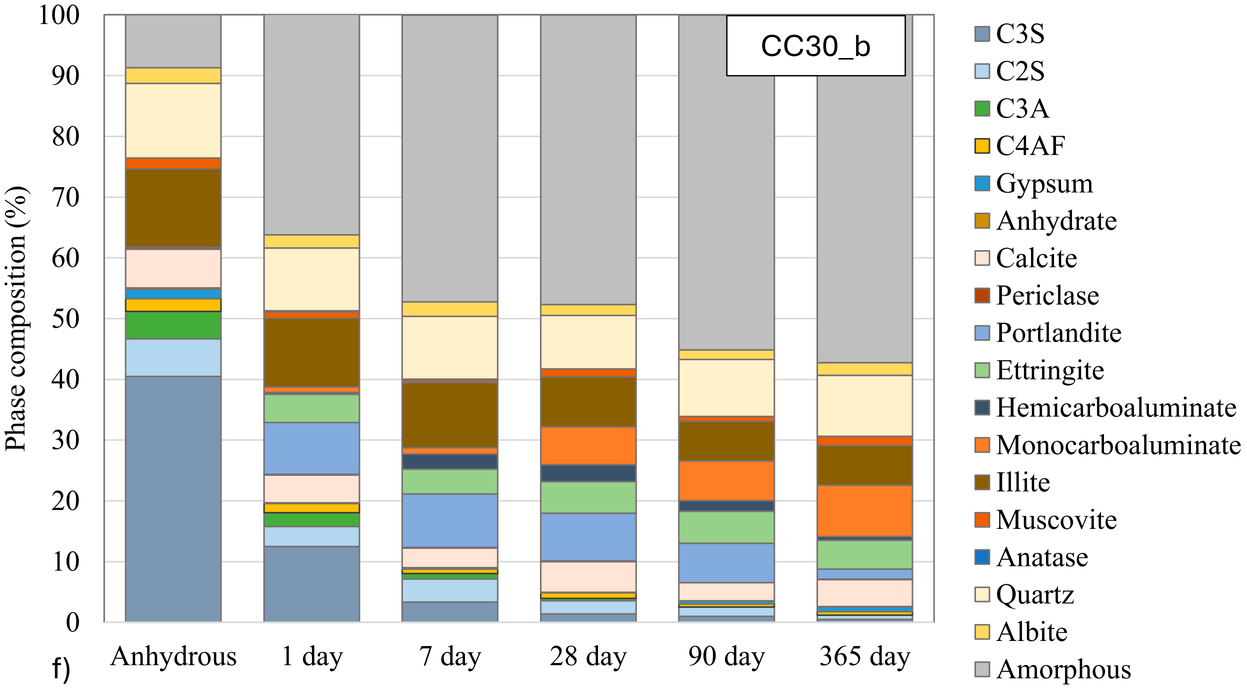

CC30_b
Phase composition (%)
100
90
80
70
60
50
40
30
20
10
0
Anhydrous
1 day
7 day
28 day
90 day
365 day
C3S
C2S
C3A
C4AF
Gypsum
Anhydrate
Calcite
Periclase
Portlandite
Ettringite
Hemicarboaluminate
Monocarboaluminate
Illite
Muscovite
Anatase
Quartz
Albite
Amorphous


f)

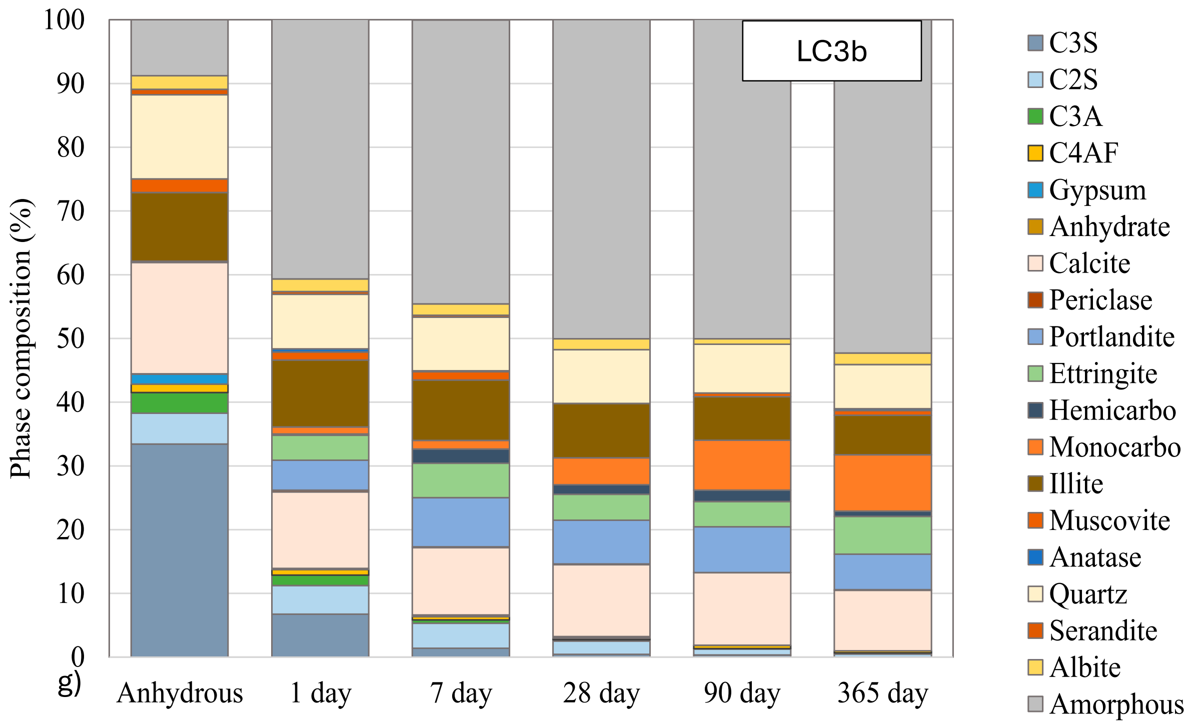


**Figure 2.** Graphical representation of XRD Rietveld quantification results of cements and cement paste normalised per 100g of anhydrous for a) OPC, b) FA30 and c) HVFA, d) CCa30, e) LC3a f) CCb30 g) LC3b.

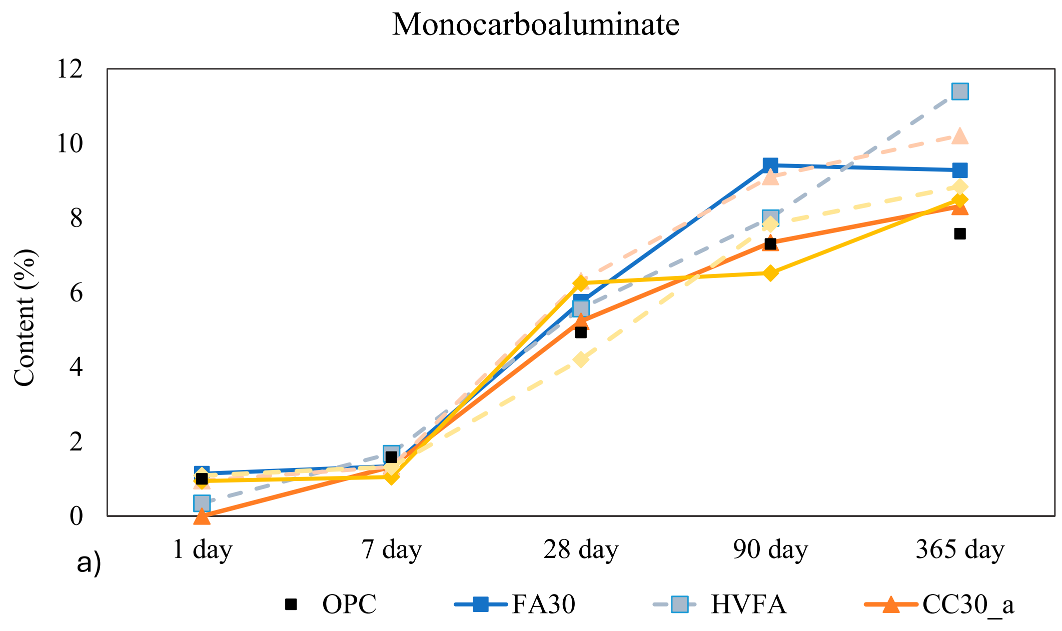

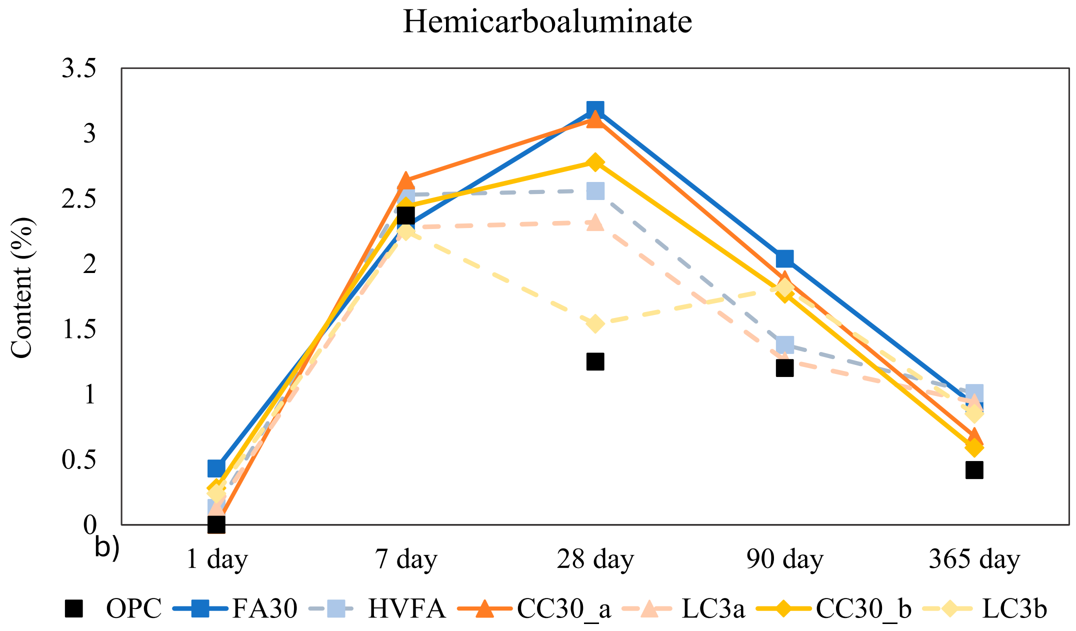


**Figure 3.** Calculated a) monocarboaluminate , and b) hemicarboaluminate content

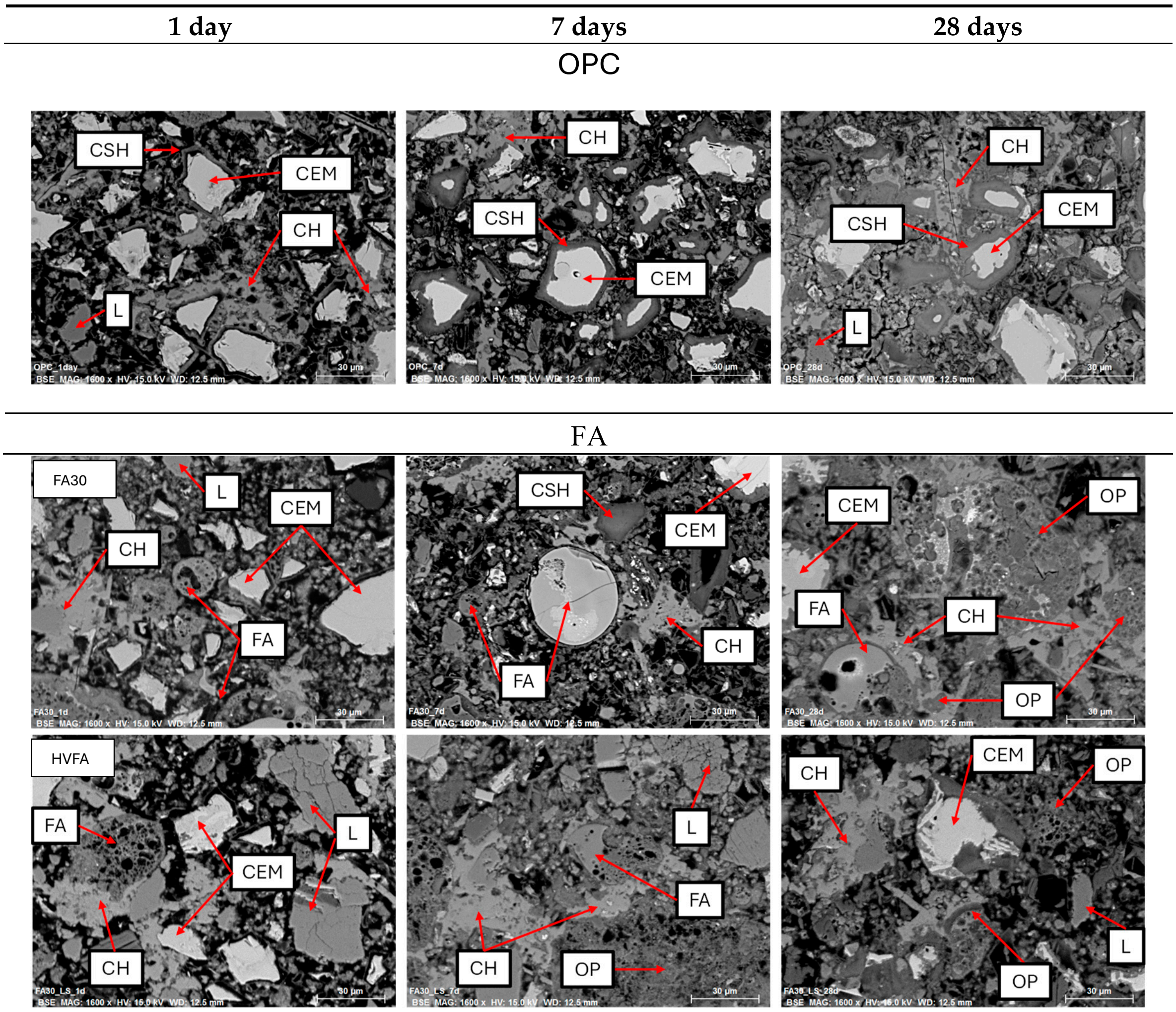

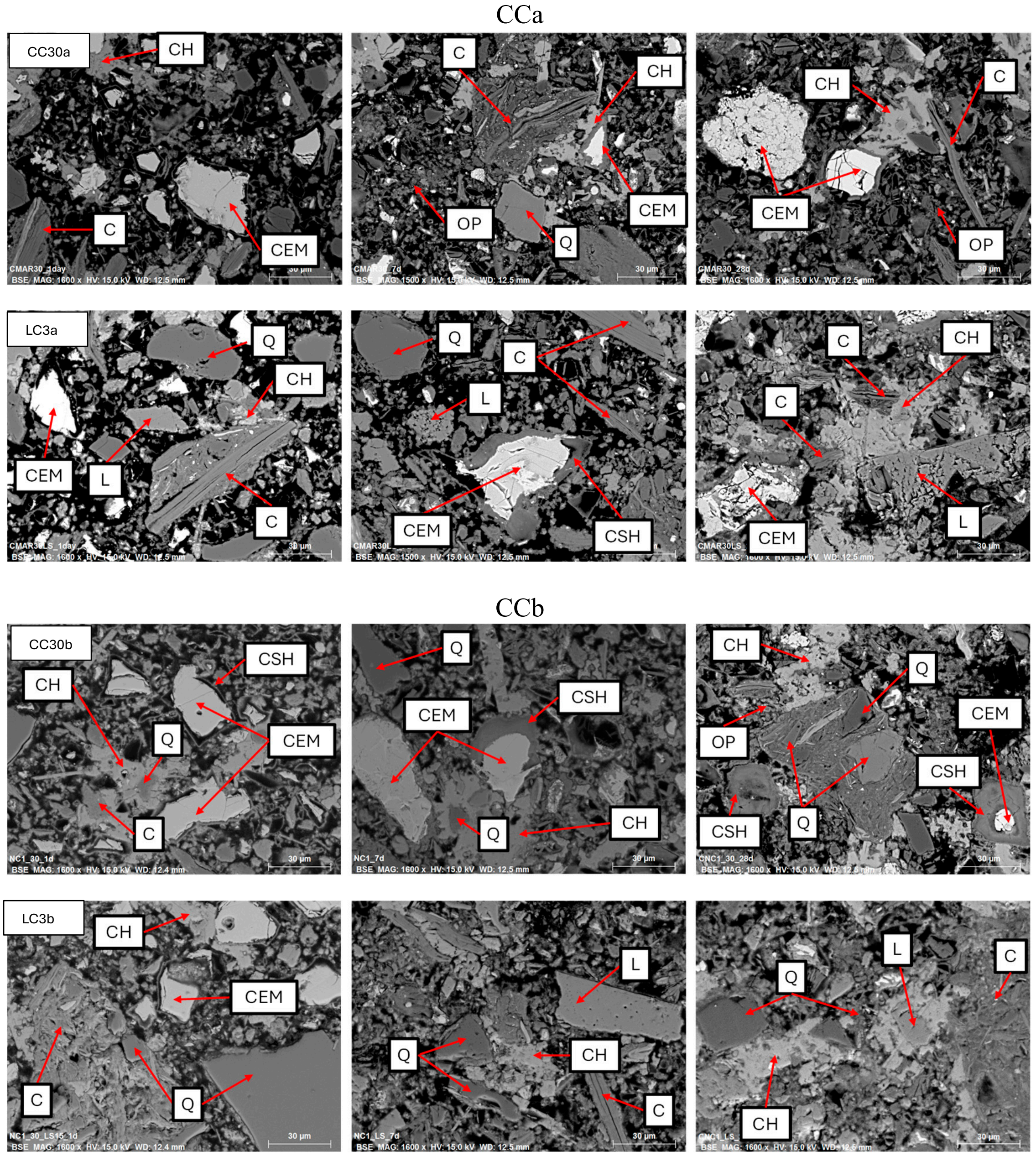


**Figure 4.** SEM BSE micrograms of OPC, binary (a) and ternary (b) binder mixes after 1, 7 and 28 days of curing.
